# As Depressing As It Was Predictable? Lung Cancer, Clinical Trials, and the Medical Research Council in Postwar Britain

**DOI:** 10.1353/bhm.2007.0012

**Published:** 2007

**Authors:** Carsten Timmermann

**Keywords:** lung cancer, smoking, surgery, radiotherapy, chemotherapy, clinical trials, ethics, Britain, Medical Research Council

## Abstract

In recent years lung cancer specialists have complained that due to stigma resulting from the association of the disease with smoking, theirs is a neglected field. This paper demonstrates that in the 1950s and 1960s, when the British Medical Research Council (MRC) started to organize clinical trials for various forms of cancer, this was not the case. Rather, the organizers of these trials saw lung cancer as a particularly promising object of research, for much was known about the disease. The cancer trials were part of a strategy to use the Randomized Controlled Trial (RCT) technology to cement the role of the MRC as the dominant body overseeing medical research in Britain. The organization of the trials, however, turned out to be very difficult, due to ethical problems and the dominance of one form of therapy, surgery. The trial results were deeply disappointing. I argue that these frustrating results contributed to the notion of hopelessness that has come to surround lung cancer, and to the shift of focus from cure to prevention that was triggered by epidemiologic studies identifying tobacco smoke as the main cause of the disease. The paper deals with an important episode in the history of clinical cancer research in postwar Britain, illustrating the ethical and practical problems faced by the organizers.

## Introduction

It has been a common complaint in recent years among lung cancer specialists that theirs is a neglected field, and that the main reason for this is a stigma resulting from the association of the disease with smoking. Claudia Henschke and Peggy McCarthy, for example, in a recent book have argued that “in part because of pervasive negative feelings about smokers (and even ex-smokers), many lung cancer patients aren’t offered the aggressive treatments routinely provided for those with other types of cancer.”^1^ In an informal conversation with this author, a leading British medical oncologist specializing in lung cancer therapy reported similar attitudes: medical researchers who wanted to undertake research on lung cancer, he suggested, always struggled for resources because finding cures and treatments for other cancers is seen as more important, for lung cancer is perceived as a self-inflicted disease. Whatever the truth of these arguments today, I argue here that such claims do not hold for Britain in the 1950s and 1960s. On the contrary, the British devoted considerable research attention to lung cancer therapy in these decades, and the failure to develop a successful treatment had more to do with technical difficulties than with any stigma associated with the disease.

Looking at a series of clinical studies of lung cancer funded by the British Medical Research Council (MRC) between the mid-1950s and the mid-1970s, I will argue that when planning for these trials started, the expectations in lung cancer treatment were not significantly different from those for other cancers. The lung cancer trials, as we will see, were part of an attempt to introduce the Randomized Controlled Trial (RCT) technology, which had been used successfully to evaluate the effectiveness of streptomycin in the treatment of tuberculosis, to clinical cancer research. While only a minority of lung cancer patients in the 1950s could expect to be cured, and there was little hope of long-term survival for the majority, there was also no general notion that lung cancer would remain incurable. Neither, as work by Charles Webster, Virginia Berridge, Paolo Palladino, and others indicates, was there much of a stigma attached to smoking in the 1940s and 1950s.^2^ Lighting up in the wrong place or at [Other P-312] the wrong time was viewed as an expression of bad manners, but smoking was seen as a legitimate pleasure (and at worst, as a minor vice). Concerns over the health of nonsmokers were rare. Risk and the long-term implications of the habit entered the debate from the mid-1950s, when the results of epidemiologic studies on smoking and health made it into the newspapers and started to inform a new agenda in public health and health education.^3^ Only since the 1970s, and, as Berridge shows, partly in response to the debates over smoking and health, has this new agenda centered on risk factors and individual lifestyle choices come to dominate public health debates in Britain.^4^

In the absence of stigma, how do we explain the apparent neglect of lung cancer research that current-day authors point to? I will argue that an explanation can be found in the history of treatment trials, whose organization was made complicated by the fact that there was a well-established treatment, surgery. This, along with the new ideal of randomization, led to ethical problems for the organizers of the trials. Could researchers withhold the option of surgery from patients if there was only a slight chance that these patients might benefit from an operation? A trial that was not ethical was also not feasible, even if it might provide interesting results. Moreover, the main groups involved with the preparation of the trials at this stage, radiotherapists and surgeons, had different opinions as to what was good practice. While the organizers of the MRC cancer trials initially considered lung cancer as a particularly suitable target for therapeutic trials, these problems, as we will see, led to trials about which hardly anybody was enthusiastic. References to the link with smoking and the perception that individuals brought the cancer upon themselves provided the clinical researchers conducting these trials with a way of dealing with the disappointing outcomes by shifting the focus of attention from cure to prevention. [Other P-313]

## MRC Trials and Lung Cancer Therapy, 1957–1973

Lung cancer therapy today certainly seems less innovative, with fewer headlines about advances than in other cancers. Hopes for new cures and research progress, which dominate debates around other forms of cancer, are often absent in discourses centering on lung cancer. However, this was not the case in the 1950s. On 31 January 1957, five months before the publication of its important “Statement on Tobacco Smoking and Cancer of the Lung,” the Medical Research Council held a Conference on the Evaluation of Different Methods of Cancer Therapy.^5^ The conference, under the chairmanship of the renowned professor of radiotherapy at Middlesex Hospital Medical School, Brian W. Windeyer, recommended that the Council “should consider undertaking an investigation into the treatment of certain tumours which appeared particularly suitable for short-term study.”^6^ They included in this group carcinoma of the bronchus, esophagus, and bladder; bone sarcoma; and medulloblastoma. Carcinoma of the bronchus was chosen “after considerable discussion” because of “the vast amount of material which was available and the existence of a good deal of confusion of thought about the best form of treatment.”^7^ It seems that a strong argument for the inclusion of lung cancer was that this was a malignant disease that was particularly well researched and understood and yet was also the focus of disagreement about the best treatment.

The notion that lung cancer was particularly well understood had its origins in the intense interest generated by the mysterious increase in the incidence of this once rare and obscure disease, which clinicians and pathologists observed to have occurred since the beginning of the twentieth century. Debates over whether this increase was real, and its possible causes, grew more intense toward the 1950s; the suspects, besides cigarette smoking, were the tarring of the roads, exposure at work, or car fumes and smog. These well-documented debates provided the background for the epidemiologic studies by Richard Doll and Austin Bradford Hill in Britain and by Evart Graham and Ernst Wynder in the United States, and for the reports by the MRC, the Royal College of Physicians (see the [Other P-314] article by Virginia Berridge in this issue), and the U.S. Surgeon General on smoking and health.^8^ While among the medical profession consensus formed fairly quickly regarding the dangers of smoking, wider debates over the meanings of smoking and the political consequences that were to be drawn from the results of these epidemiologic studies, as Berridge shows, lasted much longer. Contrary to current assumptions, however, the high profile that lung cancer gained through these debates stimulated therapeutic research.

The standard treatment for most lung cancers was (and still is) surgery. A typical lung cancer patient in the 1950s (usually a middle-aged man) would see his GP about chest problems: difficulties with breathing, or even blood in the sputum. The task of the GP was then to decide whether or not the patient was suffering from one of the “conventional” chest problems, such as TB or bronchitis. Usually, the patient would be referred to the local chest X-ray service.^9^ If a shadow was visible in the X ray, and there were no TB bacilli in the sputum, cancer was a possibility. A sputum sample would be screened for malignant cells, and the patient would be referred to a chest surgeon for a bronchoscopy.^10^ If the patient’s general condition was good and there were no metastases, the surgeon would perform an exploratory thoracotomy, a pneumonectomy (resection of a whole lung), or, increasingly, a lobectomy (resection of [Other P-315] the affected lobes).^11^ These were serious operations, which carried considerable risks, and patients frequently died from complications. A focus of innovation, therefore, apart from the operation as such, was diagnosis: the search for methods that allowed the distinction between operable and nonoperable cases. But what was to be done with the patients whose tumors were already too large for surgery at the time of diagnosis, or had metastasized or belonged to a cell type that was likely to do so? Should they simply be sent home to die? Or did new methods in radiotherapy or chemotherapy offer new means of intervention, for purposes of palliation or even cure?

The agenda set by the recommendations of the 1957 conference was heavily geared toward the evaluation of new approaches in radiotherapy, which was the form of therapy from which British cancer specialists most expected innovative impulses—in spite of disappointments with the treatment of lung cancer.^12^ This was perhaps not surprising, given the strength of the field in Britain and the strong presence of radiotherapists on the committee. The MRC had played a central part in the rise of radiotherapy in the United Kingdom in the interwar years.^13^ Radiotherapists were increasingly discontent with the key position of surgeons in the treatment of malignant disease, and with the notion that surgery was the default treatment for all patients who had any hope of survival, while radiotherapists had difficulties in recruiting trial subjects. [Other P-316]

## Randomized Trials

In June 1957 the Council appointed a Steering Committee, also chaired by Windeyer, to prepare the appropriate trials.^14^ The Steering Committee then appointed five ad hoc working parties for the chosen forms of cancer, to assist with the task of “drawing up a coordinated scheme of investigation.”^15^ A sixth working party was to be appointed to work on leukemia. The composition of all these subcommittees followed the same pattern: each included a physician, a surgeon, a pathologist, a radiotherapist, and a statistician. With the proposals of the working parties in hand, the committee recommended that

favourable consideration should be given, if possible, to the support of any suitable clinical trials in the field of cancer therapy which can be carried out without too elaborate an organisation and with reasonable promise of yielding useful information.^16^

The research program drawn up by the committee was, it seems, at least as much about the development of new methods of clinical research as about finding new cancer therapies. The committee was a vehicle for applying the new RCT approach to the evaluation of well-established and new therapeutic methods, especially in radiotherapy. The use of the new technology for establishing the effectiveness of streptomycin in the treatment of tuberculosis had provided the MRC with a much-publicized success.^17^ The timing of the 1957 conference, with the results of a number of conventional trials already published or about to be published, as well as the subsequent discussions in the Working Party, suggests that the use of the RCT may even have had priority over the development of specific new therapeutic techniques. Extending the Council’s activities to cancer research (the domain of the Imperial Cancer Research Fund and the British Empire Cancer Campaign) was part of an MRC strategy to establish the Council as the main body controlling clinical research in Britain.^18^ The RCT technology, which built on some of the key strengths [Other P-317] of MRC-funded clinical research and came to embody its ethos, was a vehicle for this strategy.^19^

The members of the ad hoc Working Party (Table 1) appointed to assist the Steering Committee (Table 2) with the organization of the trials on carcinoma of the bronchus were Dr. John Guyett Scadding, Dr. L. G.

**Table 1:** Members of the Working Party on the Treatment of Carcinoma of the Bronchus

John G. Scadding (Chairman)	Chest physician, Brompton Hospital; Dean and Director of Studies, Institute of Diseases of the Chest; Physician and Senior Lecturer in Medicine, Postgraduate Medical School, Hammersmith Hospital, London
Leslie G. Blair	Radiologist; Director of the X-Ray Department, Hospital for Sick Children, Great Ormond Street; Radiologist to the Brompton Hospital, the Harefield Sanatorium and Hospital, and St Vincent’s Hospital. Published on the diagnosis and radiological treatment of chest injuries and chest diseases.
Alphonsus L. d’Abreu	Thoracic surgeon, United Birmingham Hospitals; Reader in Thoracic Surgery, University of Birmingham; Hunter Professor, Royal College of Surgeons, 1939 and 1946
Jethro Gough	Pathologist; Professor of Pathology and Bacteriology, Welsh National School of Medicine, Cardiff. Published on pneumoconiosis in coal miners.
Austin Bradford Hill	Epidemiologist and statistician; Professor of Medical Statistics at the London School of Hygiene and Tropical Medicine
Brian W. Windeyer	Radiotherapist; Director of the Meyerstein Institute of Radiotherapy, Middlesex Hospital, and the Radiotherapy Department, Mount Vernon Hospital; Professor of Therapeutic Radiology at the University of London; Dean of the Middlesex Hospital Medical School[Other P-318]

**Table 2:** Members of the MRC Steering Committee

Brian W. Windeyer (Chairman)	See Table 1
Joseph S. Mitchell	Radiotherapist; Honorary Director of the Radiotherapy Centre at Addenbrooks Hospital, Cambridge; Regius Professor of Physic at the University of Cambridge, 1957–1975
Robert B. Hunter	Clinician; Professor of Materia Medica and Lecturer in Clinical Medicine, University of St Andrews
Robert W. Scarff	Pathologist; Director of the Bland-Sutton Institute of Pathology, Middlesex Hospital, and Professor of Pathology at the University of London; Honorary Secretary of the British Empire Cancer Campaign
Austin Bradford Hill	See Table 1
Leslie J. Witts	Hematologist; Nuffield Professor of Clinical Medicine, Radcliffe Infirmary, Oxford

Blair, Mr. A. L. d’Abreu, Professor J. Gough, Professor Austin Bradford Hill, and Professor Windeyer. The chairman, Scadding, was consultant chest physician at the Brompton and Hammersmith Hospitals and professor at the Institute of Diseases of the Chest, the specialist medical school associated with the Brompton Hospital. He is hailed as one of the founding fathers of respiratory medicine in Britain, and was one of the founders and later president of the Thoracic Society and the first editor of the journal *Thorax*.^20^ Along with Bradford Hill, who was consulted whenever the Council needed statistical expertise, he had been involved in the streptomycin trials.^21^ The Working Party recommended that the Steering Committee undertake a randomized trial of different forms of radiotherapy in a small number of centers (they explicitly mentioned Edinburgh, Newcastle, Manchester, Liverpool, the Middlesex Hospital, and Hammersmith Hospital). Reconstituted for this purpose, under the same chairman, the Working Party was to prepare and oversee the trial. [Other P-319] In 1959, Scadding’s colleague at the Brompton, the chest physician John Reginald Bignall, was appointed as secretary.^22^

## Ethics and Feasibility

The discussions among both the Steering Committee and the Working Party centered predominantly on what kinds of studies were (a) technically and (b) ethically doable. However, as it turns out, the two realms, the technical and the ethical, were difficult to keep separate. Ethical concerns, for example, were frequently raised by the prospect of randomization—an issue that, as we have heard, was central to the committee’s work. One of its members, Professor Robert W. Scarff, wondered “if strict randomisation was necessary since so many clinicians had a clear-cut impression of what was best for the patient and might feel random selection to be a little unethical.”^23^ Bradford Hill, seconded by Joseph S. Mitchell and RobertB. Hunter, argued that randomization was in fact necessary in order to detect marginal differences. To Hunter, this “raised in its train the question of feasibility again.”^24^

How were such problems to be overcome and appropriate trials organized? And why did they choose to look at lung cancer therapy? At the Steering Committee meeting on 13 January 1958, Professor Mitchell proposed to look at a trial he was undertaking in Cambridge as a model, and his statement points to one of the reasons for including lung cancer in the recommendations. It had to be made sure, Mitchell suggested, that each patient received

the best possible treatment appropriate to his particular case, and there should be the most careful clinical observation of each individual. Diagnosis, pathology and histology must be exact and unquestionable; a common type of tumour with a short natural history should preferably be studied to allow adequate numbers to be investigated in a reasonable time, a quantitative result without bias should be aimed at and criteria should be as objective as possible. His experience also showed that two forms of treatment, one new and one conventional, could be successfully compared and that randomisation was necessary for an accurate result.^25^

Another member of the committee, Professor Hunter, was concerned that “there were many forms of cancer which could not be suitably used in such an investigation because the pattern of treatment was so well established and so widely accepted that any deviation would cause ethical difficulty, and that this left free for investigation only the fringe of cases of hopeless prognosis.”^26^ Windeyer disagreed, arguing that there were cancers, such as carcinoma of the bladder, for which several forms of therapy were successfully used, but where confusion existed over the relative merits of the different treatment regimens. The committee members viewed lung cancer as particularly suitable because its high incidence and short natural history (after diagnosis) promised large numbers of trial subjects in a reasonable time.

Contrary to current notions, the members of the committee did not view carcinoma of the bronchus as exceptionally hopeless, but as “a representative problem.” They agreed that retrospective surveys could not supply the answers they were looking for. Long-term studies were too expensive (and this was where the short natural history comes in handy), but at least five years of follow-up were necessary. However, not only survival should be recorded: other parameters should also be taken into account, which might allow conclusions concerning quality of life—such as time spent out of hospital, time spent out of work, the degree of pain and disability, dyspnea, and hemoptysis. For lung cancer it was especially important, Mitchell suggested, “to evaluate the ordeal of treatment against possible benefit, and to try to decide if, in the late cases, X-ray treatment was worth while as opposed to simple palliation.”^27^ But this is where the problems started. At a meeting in 1959 the Working Party found it almost impossible to define criteria that distinguished palliation from prolongation of life, and defining criteria was an important stage in the organization of trials.^28^ It became increasingly obvious, as we will see in the following section, that ethical difficulties were not merely matters of procedure: they were integral to the whole enterprise of organizing clinical trials of cancer therapies.

## Finding a Suitable Question

Soon after the constitution of the ad hoc working parties in 1957 it became clear that it was not easy to find a suitable, well-contained question that [Other P-321] could be answered by way of an ethically acceptable clinical trial, within the remits set by the committee’s recommendations (promising, reasonably easy to organize, using randomization, and leading to further research). While the motivation for the trials partly derived from the streptomycin success, American cancer research also served as a model. P. Armitage of Hammersmith Hospital was invited to report on experiences with cooperative, multicenter trials in the United States. He also told the committee about a trial in progress at the Hammersmith comparing surgery with radical radiotherapy in operable cases, but with a very limited intake of patients; an attempt to compare different methods of radiotherapy had failed for technical reasons.^29^ Scadding suggested three problems that might fit the remits of the recommendations and were worth studying: (1) the efficacy of surgery as opposed to radiotherapy, “which was as yet an unsolved question”; (2) the efficacy of different kinds of radiotherapy; and (3) the use of chemotherapy alone or in combination with other forms of treatment. However, he did not believe that there was satisfactory evidence for the beneficial effects of chemotherapy, and therefore he did not think that an evaluation of its use was a suitable subject for an MRC trial. There were also, he argued, considerable ethical objections to a comparison of surgery and radiotherapy, for about a quarter of the patients undergoing surgery survived for five years or longer. For these and other reasons, Scadding was skeptical about the Hammersmith trial.^30^ The Working Party concluded that, while desirable, “a large-scale controlled investigation of the relative merits of surgery as opposed to radiotherapy did not appear feasible at the present time.”^31^ It was clear that the main factor that made such a study appear unfeasible was the expectation that it would be difficult to obtain the necessary cooperation of surgeons.

The discussions in the committee seemed to go in circles, and progress was frustratingly slow. Since so far only a minority of “some ten percent” of patients was considered for therapy at all, Windeyer asked, would it not be possible to study the remaining 90 percent, maybe by comparing different [Other P-322] forms of radiotherapy?^32^ Representing the surgeons, who were more interested in improving diagnosis, d’Abreu argued that what was really needed was information about the relative prognosis in different kinds of cancer of the bronchus, and also an answer to the question whether the prolongation of life by a few months by means of radiotherapy was worth it in terms of the quality of the life so gained. Also, did the linear accelerator improve quality of life more than conventional radiotherapy? Finally they came to a conclusion that nobody was really enthusiastic about, “that a comparative trial of different methods of radiotherapy might be considered in patients not primarily suitable for surgical treatment but regarded suitable for an attempt at cure by radiotherapy.”^33^ The inclusion of chemotherapy was “not thought to be practicable at the present stage of knowledge in this field.”^34^ The details of the trial, however (and this was an indicator of the increasing frustration), were to be determined by a working party of different constitution.

The Working Party continued to pursue the idea of a trial comparing the effects of super- and orthovoltage irradiation. Despite a distinct lack of enthusiasm on his part, Bignall volunteered to draft a provisional protocol for the trial. It was decided to approach Philip D’Arcy Hart of the MRC’s Tuberculosis Research Unit about coordinating the work in collaboration with the Statistical Unit, due to these units’ previous experience with controlled trials.^35^ It appears as if the Working Party hoped that they were going to be able to repeat their streptomycin success. D’Arcy Hart, initially reluctant because of staff shortages in his unit, accepted the offer and appointed a new member of staff, Dr. Joan Heffernan.^36^ Letters were written to the centers that the Working Party considered as likely participants in the study, and a joint meeting with radiotherapists was organized.

It was important for the Working Party to get a sufficient number of radiotherapists on board, so it made sense to involve them in the preparation. A meeting with twenty-nine consultant radiotherapists took place on 21 January 1961 in the Council Room of the Royal College of Surgeons in London.^37^ The chairman told those present that “considerable difficulty [Other P-323] had been encountered in making plans which would not only be ethically acceptable and feasible but which, at the same time, could produce information of value.”^38^ In preliminary consultations, the draft protocol had found little support; the purpose of this meeting, therefore, was “to find out whether the radiotherapists concerned were in agreement about the importance of the principle of controlled clinical trials and whether further agreement could be reached upon a subject worth trying and upon the methods involved.”^39^ Windeyer added that the MRC committee that appointed the various working parties “had felt that there was not enough controlled work at present and that not all of the claims which were being made for various forms of treatment could stand up to rigorous examination.”^40^ Would the radiotherapists provide the Working Party with clearer directions?

They did not. The radiotherapists, too, were unenthusiastic about the protocol. Most thought that, clearly, supervoltage was to be preferred to orthovoltage therapy.^41^ Dr. Tudway from Bristol spoke for many when he remarked that “it was difficult for those with a choice of treatments to believe that it was not better to use supervoltage if this was available.”^42^ Some of the radiotherapists doubted whether a trial in carcinoma of the bronchus made much sense in the first place. Ralston Paterson from Manchester conceded that “some difficulties were implicit in random selection, but he hoped that radiotherapists would encourage the Medical Research Council to continue to organize a trial”; however, he suggested that “lung cancer was one of the more difficult fields for investigation as overall mortality is high and it is difficult to assess differences in response.”^43^ Others reinforced the questions that members of the Working Party had already raised about withholding treatment from patients who might benefit from it. Many suggested, instead, that a trial should be designed to “compare the progress and survival rate of patients with presumed undifferentiated carcinomas of the lung following surgical treatment, with that following radiotherapy.”^44^ The Working Party followed their suggestions, and in the next section we will look at the results. [Other P-324]

## Trial I: Radiotherapy versus Surgery, 1961–1973

Four years after the decision to organize lung cancer treatment trials, the at times frustratingly slow negotiations over the details of these trials appeared finally to draw to a conclusion. Another meeting was scheduled with both consultant surgeons and radiotherapists on 25 July 1961.^45^ Scadding introduced the agenda by stating that “there appeared to be a clinical problem as to the right advice to give a patient with a histological report of an undifferentiated carcinoma of the bronchus—whether to advise surgery or radical radiotherapy.”^46^ Defining the problem in this way helped to overcome ethical problems: “For those who honestly felt they did not know which treatment to advise there were no ethical difficulties. If there were enough people with this doubt in their minds, the trial could be conducted.”^47^ As mentioned above, a comparison of surgery and radiotherapy was already the subject of a smaller trial at Hammersmith Hospital, but it had been difficult to find enough patients in the more than six years that the trial was running.^48^ Dr. Gwen Hilton at University College Hospital had also reported results with radiotherapy in a small number of cases that were “apparently as good as surgery.”^49^ The discussion with the surgeons, moreover, indicated that there was indeed disagreement: while some saw it as proven that resection, where possible, was always superior to other forms of treatment, others argued that for this kind of tumor it was time to move away from surgical treatment whose results were uniformly poor, and to turn to radiotherapy or chemotherapy. In most places, according to one radiotherapist (Dr. Fleming, St. Thomas’s), only “surgical rejects” were treated with radiotherapy.^50^

However, while the agenda was set, there were still difficulties. A central problem was the eligibility of patients for the study. After the consultation with the radiotherapists, the Working Party had returned to an option for a trial design that its members had dismissed at an earlier stage of [Other P-325] the discussion, but that they now gave a specific focus on what at this stage they described as either anaplastic or undifferentiated carcinoma of the lung.^51^ Restricting eligibility on grounds of cell type made a study feasible that originally was unacceptable on ethical grounds to some members of the Working Party. But tumor grading was a difficult business. One of the surgeons present at the meeting with the Working Party (Mr. Nohl, Harefield) pointed out that in his experience nearly one-fifth (18 percent) of histological reports were mistaken.^52^ Grading schemes had changed significantly since the first attempts to classify tumor cells in the nineteenth century, with new techniques and pathological material becoming available. Later, new forms of therapy encouraged further distinctions between cell types: some tumors proved to be more susceptible to certain treatments, which made distinctions meaningful that had not carried any meaning before.^53^ In the early 1960s, some “undifferentiated” or “anaplastic” bronchial tumors were reclassified as carcinomas of small-cell or oat-cell type.^54^ This reclassification exercise was partly driven by experiences with chemotherapy (small-cell carcinomas are very responsive to chemotherapy) and partly by attempts to establish an internationally consistent terminology. During discussions, the different terms were used interchangeably. By the time the first results were published in 1966, the trial was described as “Comparative Trial of Surgery and Radiotherapy for the Primary Treatment of Small-Celled or Oat-Celled Carcinoma of the Bronchus.”^55^

The results of the trial were not encouraging: after two years, only 3 of the original 71 surgical patients and 10 of the 73 radiotherapy patients were still alive. According to the report in the *Lancet*,

the number of survivors at 24 months is so small that further statistically significant differences between the series in this respect cannot now arise. Despite the [Other P-326] slightly higher proportion of short-term and long-term survivors in the radical radiotherapy series both policies have produced very poor results in this highly malignant form of carcinoma, confirming the findings in other series.^56^

The working party suggested that radiotherapy might be the slightly better choice, since postoperative complications would be avoided.

However, because the results of the treatment are so poor whether by surgery or radical radiotherapy there is an urgent need for further research to improve the treatment of this condition. There is also an urgent need to apply the knowledge already available, in particular that of the role of cigarette smoking . . . to the *prevention* of the disease.^57^

We can see how in the light of widening acceptance of the tobacco hypothesis the focus is shifting toward prevention, in line with what Berridge observes for policy formation. A note in the administrative file dealing with the study states: “It seems to me that there is nothing at all controversial about this report, which is a straightforward account of a difficult but well organized clinical trial, the outcome of which has been as depressing as it was predictable.”^58^ Nevertheless, follow-up for the thirteen survivors continued as planned. After five years, only one surgery patient and three in the radiotherapy group were alive, and after ten years the last surgery patient had died (this patient, while originally assigned to the surgery group, had become too breathless to withstand an operation and received palliative radiotherapy instead—and he was not the only member of this group who turned out to be inoperable when surgery was scheduled). The three survivors in the radiotherapy group were still alive and well after ten years.^59^

One official goal of this and other clinical trials was to provide evidence that would lead to closure in a controversy. The debate unfolding on the letter pages of the *Lancet* after the publication of the first report indicates that this was not achieved. The study was criticized by leading specialists such as Roger Abbey Smith of the Thoracic Unit at the King Edward VII [Other P-327] Memorial Chest Hospital in Warwickshire, who argued that the reason why the results for surgery were so bad was that only patients with particularly unsuitable, centrally located oat-cell tumors were included in the study. Nine of the 71 patients in the series were not operated on because their condition deteriorated too rapidly, and of 58 patients where exploratory surgery was performed, 24 were found to be inoperable. In all patients whose tumors were resected, this was done by pneumonectomy, a type of operation that posed greater risks to the patients than the less radical lobectomy. Abbey Smith argued that the results for surgery would have looked much better had peripheral tumors also been included, and the Working Group could therefore claim validity of these results only for centrally located oat-cell carcinomas.^60^ John Rashleigh Belcher, another leading specialist, based at the Middlesex Hospital, doubted if the conclusion that radiotherapy was superior to surgery in this situation was valid, for the numbers of patients were small and only the patients who were really operated on should have been included in the statistics for the surgery group.^61^ This was seconded by Kent Harrison (St. Thomas’s), who argued that there was a risk that uncritical readers might now accept that surgery had no place in the treatment of small-cell carcinoma, which was clearly not the case. He believed that any apparently operable carcinoma of the lung should be operated on, no matter what the cell type, and for this reason he had not participated in the trial.^62^ Surgeons still criticized the trial in the 1980s for the image of hopelessness they thought it had created around lung cancer surgery, especially for oat-cell carcinoma.^63^

Scadding defended the study that his Working Party had organized, arguing that, even taking these criticisms into account, the results were not significantly different and the outlook remained bleak:

The facts should be publicised: the incidence of a disease which has assumed epidemic proportions, which has a high mortality, and for which no current method of treatment can be regarded as satisfactory, would be reduced to a small fraction of its present level if men and women as responsible individuals chose to give up, or never to take up, cigarette smoking.^64^

It seems that the frustrating outcomes of a trial about which nobody was very enthusiastic in the first place reinforced an ongoing shift of focus from therapy to the prevention of lung cancer. However, by presenting experiences with the treatment of small-cell carcinoma, an especially malignant type of cancer, as representative of lung cancer more generally, it may be argued that Scadding made the outlook for lung cancer patients seem even bleaker than it may have been anyway.

## Trial II: Adjuvant Chemotherapy, 1964 –1976

The second trial overseen by the Working Party was a study of chemotherapy as an adjuvant to surgery. The trial started in 1964, after having been discussed at a Working Party meeting in 1963. Like the first trial, this second one was also coordinated by the MRC Tuberculosis Research Unit. In charge of both trials at the Unit was Anthony Bernard Miller, replacing Joan Heffernan.^65^

The preparation of the chemotherapy trial, it seems, was much smoother than that of the first trial: there were no extensive debates in the Working Party, and no big meetings with consultants. One explanation for this lack of controversy may be that chemotherapy was tested only as a secondary therapy, an adjuvant to surgery, to prevent the growth of secondary tumors. It may also be due to the fact that in chemotherapy (unlike radiotherapy) there were few entrenched positions. It was perceived as something new, an approach that promised new channels for intervention (and also something that the British were not particularly good at and still had to learn a lot more about).^66^

Patients were randomly assigned, in a double-blind set-up, to groups that were prescribed either a placebo or one of two chemotherapeutic agents, busulphan or cyclophosphamide.^67^ The drugs were prepared as tablets, to be taken at home when the patients were discharged after [Other P-329] their operations. Finding participants does not seem to have been too difficult: Miller’s satisfied letters to the collaborators report on steady progress with patient intake. By 10 March 1965, fourteen surgeons and thirty-four chest physicians had declared that they were taking part.^68^ By 26 July 1965, 122 patients had been admitted to the study.^69^ According to Miller, the study was “running very smoothly from an organizational point of view and [would] clearly yield answers to the questions originally posed in the protocol.”^70^ There were, however, some unexpected problems with the toxicity of the drugs, and the dosage had to be reduced in December 1965. By February 1968, there were 749 patients in the study, and the Working Party decided that the intake could be stopped.^71^ The final intake was 753 patients in twenty-three centers throughout the U.K. These were centers where chest physicians and surgeons had agreed to cooperate, and where patients could be transferred to the care of the chest physician after discharge from hospital.

The only other difficulty the organizers encountered, besides the unexpectedly high incidence of hazardous toxicity with busulphan in the early stages of the study, was one that is very common to treatment trials: the problem of ensuring that patients took the right number of tablets, not more and not fewer than they had been prescribed. The organizers suggested that patients should occasionally receive home visits by health visitors, who on these occasions could count the patient’s remaining tablets.^72^

While the Working Group had shown that it was able to organize a clinical study in lung cancer that conformed to the new standards of a cooperative, double-blind, randomized controlled trial, the results did not fulfil its expectations: “The therapeutic results at two years are disappointing, for there is no evidence that either of the two cytotoxic drugs in the dosage used improved survival.”^73^ After five years, 27 percent of the patients who received cyclophosphamide were still alive, 28 percent of those on busulphan, and 34 percent in the placebo group.^74^

## Conclusion

Let us disregard the disappointing results of these trials for a moment. Judging from the attention that lung cancer received from the Medical Research Council in the 1950s and 1960s, it appears that this was not a particularly neglected form of malignant disease. Indeed, the remarkable increase in incidence also triggered interest among researchers, with a view to not only lung cancer etiology, leading to the work that linked this disease firmly with cigarette smoking, but also its treatment.^75^ Explicitly because they considered it a well-researched form of cancer on which much was known, the members of the MRC Steering Committee in 1957 wanted to see studies on lung cancer included in the program of randomized controlled trials that they were preparing.

Nor did this interest disappear quickly. In 1979, still, about 10% of cancer treatment studies then under way in Britain were dealing with lung cancer, 21 studies out of 211 for which questionnaires were returned.^76^ This compared to 49 trials concerned with breast cancer, 30 with lymphomas, and 25 with leukemia. However, the absolute figures may be slightly misleading: with an estimated total incidence of nearly 32,000 lung cancer cases per year, this meant that only 2% of the patients were entered into trials, compared to 8% of breast cancer patients, 9% of those diagnosed with melanoma, 17% of those suffering from Hodgkin’s lymphoma, and an impressive 27% of lymphoblastic lymphoma patients.^77^

What makes the treatment of one form of malignant disease a more interesting and rewarding subject for research than another? As Ilana Löwy, Jean Paul Gaudillière, and others have shown, for blood and lymph cancers it was partly the convergence of interest between cell biologists and cancer researchers.^78^ Moreover, while the results of most studies on [Other P-331] treatments for lung cancer were disappointing, in childhood leukemia (and this is the other extreme) it was increasingly discussed in the 1960s and 1970s whether it was not appropriate, after the highly visible, successful trials with new regimes of multidrug chemotherapy, to talk about cure rather than temporary remission.^79^ Increasingly this approach to cancer therapy was seen as a model that clinical researchers sought to apply to other forms of malignant disease.^80^ In lung cancer, in contrast, the only therapy that could really be expected to bring any prospect of longer-term survival was (and still is) surgery, following increasingly routinized pathways that separated operable from nonoperable patients.

Bronchial carcinoma turned out to be a particularly “recalcitrant” form of malignant disease. By the time Wallace Fox and Scadding published the ten-year follow-up results of the MRC trial of surgery and radiotherapy, carcinoma of the bronchi was by far the commonest malignant tumor in Britain and most other developed countries, and was still increasing in frequency. Its mortality, moreover, was little affected by treatment. A *Lancet* editorial in 1975 suggested that the outlook for sufferers was dire: the proportion of all patients “diagnosed and treated with the best means available” who could expect to survive for five or more years was estimated to be one in twenty in the late 1950s, and this had not changed significantly in the meantime.^81^ Technically satisfactory resection was possible in about a quarter of cases and accounted for most of the long-term survivors, most of whom had squamous-cell carcinomas. Radiotherapy occasionally led to long-term survival, especially with oat-cell (or small-cell) carcinomas, and it was useful as a palliative treatment. Attempts to use cytotoxic chemotherapy had so far proved disappointing, especially with a view to the considerable impact this form of treatment could have on quality of life. Screening, too, did not seem to deliver any significant benefits.

In the light of these results, the authors of the editorial concluded that “the overwhelming importance of a preventive approach to this disease must always be emphasised. It would be a cruel deception to allow smokers to think that any improvement in treatment, or any procedure to which they might submit themselves to attain early diagnosis, is likely to diminish appreciably their risk of dying of lung cancer.”^82^ If there is [Other P-332] really, as Henschke and McCarthy argue, a lack of interest in research on lung cancer today, the remarkable recalcitrance of this group of malignant diseases is at least partly to blame. The stigma that these authors project back into the past may be a result rather than a cause. In the crucial period up to the mid-1970s, the application of the innovative techniques that made other cancers interesting to researchers, funding bodies, and companies, in clinical trials that were feasible and ethically acceptable (criteria that were difficult enough to fulfil, as we have seen), simply did not seem to make much of a difference for lung cancer patients. It appears that it was partly frustration that has made lung cancer less visible than other cancers.

The apparent neglect of bronchial carcinoma in clinical research was not caused by stigma, as I have shown. Rather, the notion of hopelessness in lung cancer therapy and the stigma of the self-infiicted disease emerged around the same time. The tide may be turning, though. Books like that by Henschke and McCarthy, charities dedicated specifically to lung cancer (like the Roy Castle Foundation in Britain), and the mere fact that medical oncologists are specializing in lung cancer research may all be indicators of change. In a climate where smoking is increasingly medicalized and viewed as an addiction rather than a matter of choice, depicting lung cancer as a neglected field may be a good strategy for generating attention and increasing its visibility. [Other P-333]
